# 2-Bromopropionyl Esterified Cellulose Nanofibrils as Chain Extenders or Polyols in Stoichiometrically Optimized Syntheses of High-Strength Polyurethanes

**DOI:** 10.1021/acs.biomac.2c00747

**Published:** 2022-10-06

**Authors:** Mengzhe Guo, You-Lo Hsieh

**Affiliations:** Biological and Agricultural Engineering and Chemical Engineering, University of California at Davis, Davis, California95616-8722, United States

## Abstract

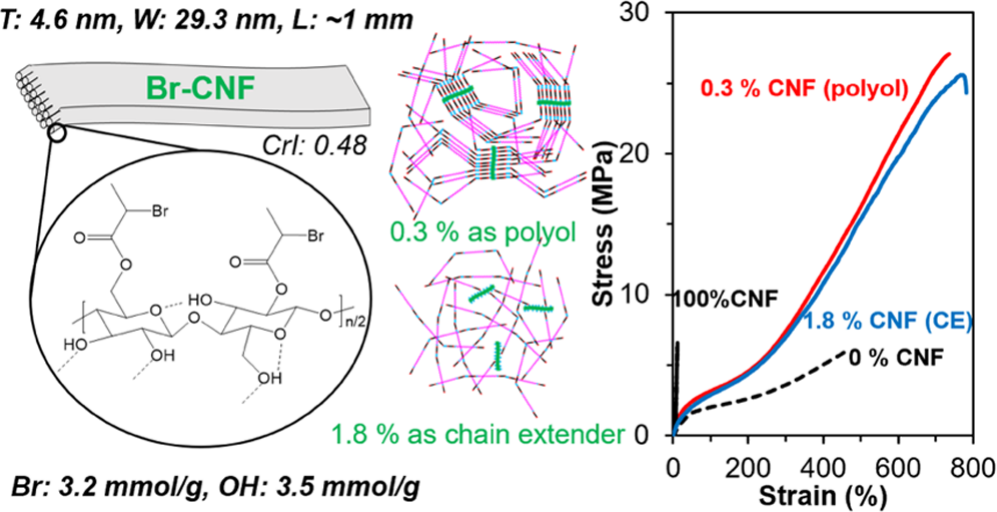

2-Bromopropionyl bromide esterified cellulose nanofibrils (Br-CNFs) facilely synthesized from one-pot esterification of cellulose and in situ ultrasonication exhibited excellent *N*,*N*-dimethylformamide (DMF) dispersibility and reactivity to partially replace either chain extender or soft segment diol in the stoichiometrically optimized syntheses of polyurethanes (PUs). PUs polymerized with Br-CNF to replace either 11 mol% 1,4-butadiol chain extender OHs or 1.8 mol% polytetramethylene ether glycol OHs, i.e., 1.5 or 0.3 wt% Br-CNF in PUs, exhibited an over 3 times increased modulus, nearly 4 times higher strength, and a 50% increase in strain. In either role, the experimental modulus exceeding those predicted by the Halpin–Tsai model gave evidence of the stoichiometrically optimized covalent bonding with Br-CNF, while the improved strain was attributed to increased hydrogen-bonding interactions between Br-CNF and the soft segment. These new Br-CNFs not only offer novel synthetic strategies to incorporate nanocelluloses in polyurethanes but also maximize their reinforcing effects via their versatile polyol reactant and cross-linking roles, demonstrating promising applications in the synthesis of other polymers.

## Introduction

Cellulose nanocrystals (CNCs) and cellulose nanofibrils (CNFs) are attractive one-dimensional nanofillers to reinforce polymer matrixes due to their intrinsically high surface area,^1^ high Young’s modulus (150 GPa for CNCs, 28 GPa for CNFs),^2,3^ and very low coefficient of thermal expansion (10^–7^ K^–1^ for CNCs, 5 × 10^–6^ K^–1^ for CNFs).^4,5^ CNCs are most commonly obtained by removing the noncrystalline domains in cellulose by sulfuric acid hydrolysis,^6−10^ whereas CNFs are derived by separating along the less crystalline domains by mechanical forces,^10−19^ chemical reactions, such as (2,2,6,6-tetramethylpiperidin-1-yl)oxyl (TEMPO) oxidation,^10−15,20^ or a combination of both.^10−15^ All these processes produce hydrophilic and, in many cases, anionically charged nanocelluloses^6−20^ that are easily aqueously dispersed and miscible with water-soluble polymers. For instance, mixing CNC^21,22^ and CNF^23,24^ with waterborne polyurethane (WPU) has produced 3.5-^21^ or 4-fold^23^ stronger films, but with considerably reduced strain at ca. 80 and 40%, respectively. The poor corrosion resistance^25^ and relatively low strength^26^ of WPU along with very high loadings of 30% CNC^21^ and 20 % CNF,^23^ however, limit their use. In the case of polyester-based thermoplastic polyurethane (TPU), mixing up to 1^27^ or 30 %^28^ CNC with the aid of ultrasonication in *N*,*N*-dimethylformamide (DMF) did not improve tensile strength or strain significantly, likely due to the incompatibility between the hydrophilic CNC and hydrophobic TPU matrix.

Incorporation of micrometer-scale microcrystalline^29^ or microfibrillated cellulose^30^ into polytetramethylene ether glycol (PTMEG, *M*_n_: 1000 Da) in the reaction with methylene diphenyl diisocyanate (MDI) and then chain extension with 1,4-butadienol (1,4-BD) were effective in increasing the tensile strength of the resulting TPUs by 3^29^ or 4.5^30^ times, respectively (Table 1). However, dispersing aqueous CNCs (0.5 wt %) in PTMEG (*M*_n_: 1000 Da) by homogenization and water evaporation followed by sequential reactions to MDI and 1,4-BD only improved tensile strength by 40%.^31^ Additional pretreatments of either freeze-drying CNC^32^ or sequential solvent exchanging CNC^33^ and CNF^34^ via acetone to DMF, followed by ultrasonication with PTMEG^32,34^ or hydroxylated soybean oil^33^ have shown to improve some of the tensile moduli,^32,33^ strength,^32−34^ and strain,^32,34^ all optimized at 1 wt % addition. In all of the above cases, a constant 2:1 NCO (MDI) to OH (PTMEG)^29−32,34^ or 1.2:1 NCO (MDI) to OH (hydroxylated soybean oil)^33^ molar ratio was used without considering nanocellulose surface hydroxyls. In addition, the shear force employed in mixing, such as homogenization^31^ and ultrasonication,^35^ can potentially reduce cellulose crystallinity by 40 and 20 % as reported on sugarcane bagasse and apple pomace,^35^ respectively.

**Table 1:** Summary of Microscale Celluloses and Nanocelluloses in PU Synthesis with 2:1 MDI/PTMEG and Chain Extension with 1,4-BD,^29−32,34^ Except for the One with 20 mol% Excess MDI and No Chain Extender (CE)^33^^,^^a^

micro/nanocellulose	cell (wt %)	cell OH content (mmol/g)	preparation	media	dispersing method	prepolymer precursor	modulus (MPa)	strength (MPa)	strain (%)	optimal cell content (wt %)	MDI (wt %) of PU
MCC^29^	1–10	NA	none	DMF (0.3 wt % LiCl)	none	PTMEG (1000)	4.9–21.1	8.0–24.0	390–970	5	31.5
							4.3×	3×	2.5×		
MFC^30^	0.5–5	1.25	none	DMF	none	PTMEG (1000)	1.2–27.7	5.8–26.3	761–1387	1	31.5
							23×	4.5×	1.8×		
CNC^31^	0.5 (0–3 mol% excess MDI)	NA	none	PTMEG (1000)	homogenization (rotor–stator homogenizer)	PTMEG (1000)	16.6–16.1	41.7–58.3	1096–1011	0.5	35.8
							1×	1.4×	0.9×		
CNC^32^	0.5–5	2.8	freeze-drying	DMF	sonication*	PTMEG (1000)	8.2–44.9	7.5–61.5	751–994	1	31.5
							5.5×	8.2×	1.3×		
CNC^33^	0.5–2	NA	solvent exchange to acetone	hydroxylated soybean oil (Agrol 3.6)	sonication (IKA T 18 Basic Ultra-Turrax)	hydroxylated soybean oil (Agrol 3.6)	11.3–45	2.5–5.3	N/A	1	35.0
							4×	2.1×			
TEMPO- CNF^34^	0.5–2	NA	solvent exchange to acetone	DMF	sonication (VCX 500 Ultrasonic Processors, 300 W,1 min)	PTMEG (2000)	8.7–4.9	4.5–39.8	419–2344	1	19.5
							0.6×	8.8×	5.6×		

a Tensile property improvement was indicated by times of increases (×). * Condition not specified.

CNCs^36−59^ and CNFs^60−62^ have been esterified to convert the cellulose OHs to alkyl bromines to improve their dispersity in organic liquids, such as DMF,^36−49,59^ dimethyl sulfoxide (DMSO),^50,51,62^ tetrahydrofuran (THF),^52,53,61^ anisole,^54−57,60^ and toluene.^58,59,62^ Recently, 2-bromopropanoated nanofibrils (Br-CNFs) have been directly produced by facile one-pot esterification of cellulose with 2-bromopropionyl bromide (BPB) to 2-bromopropanoated cellulose (Br-cell) to enable disintegration by in situ ultrasonication in the same organic liquid, DMF.^63^ 2-Bromopropionyl esters on the surfaces of Br-CNF makes them compatible to DMF meanwhile the remaining surface OHs are available for reaction, enabling Br-CNF to be dual and even multifunctional from the perspective of organic reaction polymer synthesis. In the synthesis of PU, the residual surface OHs on Br-CNFs present multiple hydroxyl groups as in polyols to potentially replace diols for chain extension of diisocyanate-capped prepolymers or to replace some of the diols in prepolymer synthesis. This reaction-based incorporation of Br-CNFs in the synthesis of PU is expected to achieve the highest reinforcing effect by maximizing covalent bonding.

In this study, Br-CNFs were incorporated in the synthesis of PU in two roles. As chain extenders, Br-CNFs were added to replace some of 1,4-BD to react with MDI-capped PTMEG prepolymers. The reinforcement effects of Br-CNFs as chain extenders were investigated by replacing up to 35 mol % OHs in 1,4-BD with the surface OHs from Br-CNFs, which is equivalent to up to 5.4 wt% Br-CNFs in the PU/CNF composites. As polyols, Br-CNFs were incorporated to partially replace equal OH from PTMEG diols, both capped by MDI and then extended by 1,4-BD. The multiple surface OHs on Br-CNFs, along with their crystalline core, justify their low up to 0.5 wt% quantities. A higher molecular weight PTMEG (*M*_n_: 2900 Da), nearly up to 3 times the most commonly reported (*M*_n_: 1000^29,30,32^ or 2000^34^ Da), was selected as a soft segment to effectively lower the quantity of diisocyanate, the most toxic constituent in PU syntheses. Furthermore, MDI, with one three-hundredth vapor pressure of the more volatile toluene diisocyanate (TDI),^64^ was used as a hard segment to minimize inhalation exposure. Most significantly, not only the polyurethane reaction stoichiometry of OH_diol+Br-CNF_/NCO_MDI_/OH_diol+Br-CNF_ was rationalized but the surface OHs of Br-CNFs were also fully accounted for to optimize both prepolymer syntheses and chain extension reactions. The effective role of Br-CNFs, their molar or mass compositions, and the optimal reaction conditions, were evaluated by their effects on the elastic modulus, tensile stress, and strain-to-failure of the synthesized PU/CNF composites. The formation of the urethane link was verified by attenuated total reflection (ATR). Thermal properties, such as glass transition temperature (*T*_g_) and melting temperature (*T*_m_), of the synthesized PU/CNF film were characterized by differential scanning calorimetry (DSC). The bulk morphology and organization of Br-CNFs in the PU matrix were observed by optical microscopy under cross-polar mode.

## Experimental Section

### Materials

Cellulose was isolated from rice straw (Calrose variety) by a previously reported three-step process of 2:1 v/v toluene/ethanol extraction, acidified NaClO_2_ (1.4 %, pH 3–4, 70 °C, 5 h) delignification, and alkaline hemicellulose dissolution (5 % KOH, 90 °C, 2 h).^65^ 2-Bromopropionyl bromide (BPB, 97 %, Alfa Aesar), 4-dimethylaminopyridine (DMAP, 99 %, Acros Organics), polytetramethylene ether glycol (PTMEG, *M*_n_: 1000 and 2900 Da, Sigma-Aldrich), methylene diisocyanate (MDI, 97%, Sigma-Aldrich), 1,4-butanediol (1,4-BD, 99 %, Alfa Aesar), *N*,*N*-dimethylformamide (DMF, certified grade, Fisher Scientific), and acetone (histological grade, Fisher Scientific) were used as received without further purification. All nanocellulose concentrations in DMF were denoted in weight/volume percent (w/v%) whereas all PU/CNF compositions were designated in weight/weight percent (wt%).

### Synthesis and Characterization of Br-CNFs

Br-CNFs were produced from rice straw cellulose by one-pot esterification with 2-bromopropionyl (5:1 BPB to anhydroglucose or AGU molar ratio, 23 °C, 6 h) and in situ ultrasonication (Qsonica Q700, 50/60 Hz, 50% amplitude, 30 min) in DMF.^63^ For imaging by atomic force microscopy (AFM, Asylum-Research MFP-3D), Br-CNF DMF dispersion was diluted (10 μL, 0.0005 w/v% and deposited on freshly cleaved highly oriented pyrophoric graphite (HOPG) and then air-dried in a fume hood for 6 h. The heights of Br-CNFs (*n*: 100) were profiled in the tapping mode with a 5 μm × 5 μm scan size and a 512 Hz scan rate. For imaging by transmission electron microscopy (TEM, Philip CM12), Br-CNF dispersion (10 μL, 0.01 %) was deposited onto glow-discharged carbon-coated TEM grids (300-mesh copper, Formvar/carbon, Ted Pella Inc., Redding, CA), blotted with a filter paper after 5 min to remove excess dispersion, negatively stained with aqueous uranyl acetate (2 w/v%) for 5 min, and blotted again to remove excess liquid. This staining–blotting process was repeated five times, dried under ambient conditions for 15 min, and then imaged at a 100 kV accelerating voltage. The widths and lengths of over 100 Br-CNFs for each sample were calculated using an ImageJ analyzer (ImageJ, NIH). The crystallinity and domain size of the air-dried Br-CNF film were determined using X-ray diffraction (XRD) as described^10,63,66^ previously.

The Br content of Br-CNF (σ_Br_, mmol/g) was determined by the mass gain of 2-bromopropionyl esterified cellulose in which the C2, C3, and C6 OHs were converted to 2-bromopropionyl ester
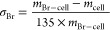
1where *m*_cell_ is the initial cellulose mass (g), *m*_Br-cell_ is the dry mass (g) of 2-bromopropionyl esterified cellulose, and 135 (g/mol) is the molecular mass difference between 2-bromopropionyl ester and hydroxyl. The substitution of surface OHs to 2-bromopropionyl, described as a fraction of converted OHs (ρ), was estimated via solution-phase ^1^H NMR (Bruker AVIII 800 MHz ^1^H NMR spectrometer) following the previously established method^63^ briefly described in the Supporting Information. Surface OH content (σ_OH_, mmol/g) of Br-CNF was calculated by multiplying Br content (σ_Br_, mmol/g) by available OH (1 – ρ) and then dividing by converted OH (ρ)
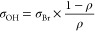
2

### Polyurethane Synthesis

The polyurethane control was prepared by dissolving MDI (1.90 mmol, 0.47 g) and PTMEG (*M*_n_: 2900 Da, 0.86 mmol, 2.5 g) in DMF (20 mL), degassed (Branson 2510) for 1 min, purged with N_2_ for 10 min, and then reacted at 90 °C in an oil bath under stirring for 3 h to form a prepolymer. The chain extender 1,4-BD (0.86 mmol, 0.078 g) was added to react at 90 °C for another 3 h and then quenched in an ice bath to end the reaction.

### Br-CNF as an Extender

**Scheme 1:**
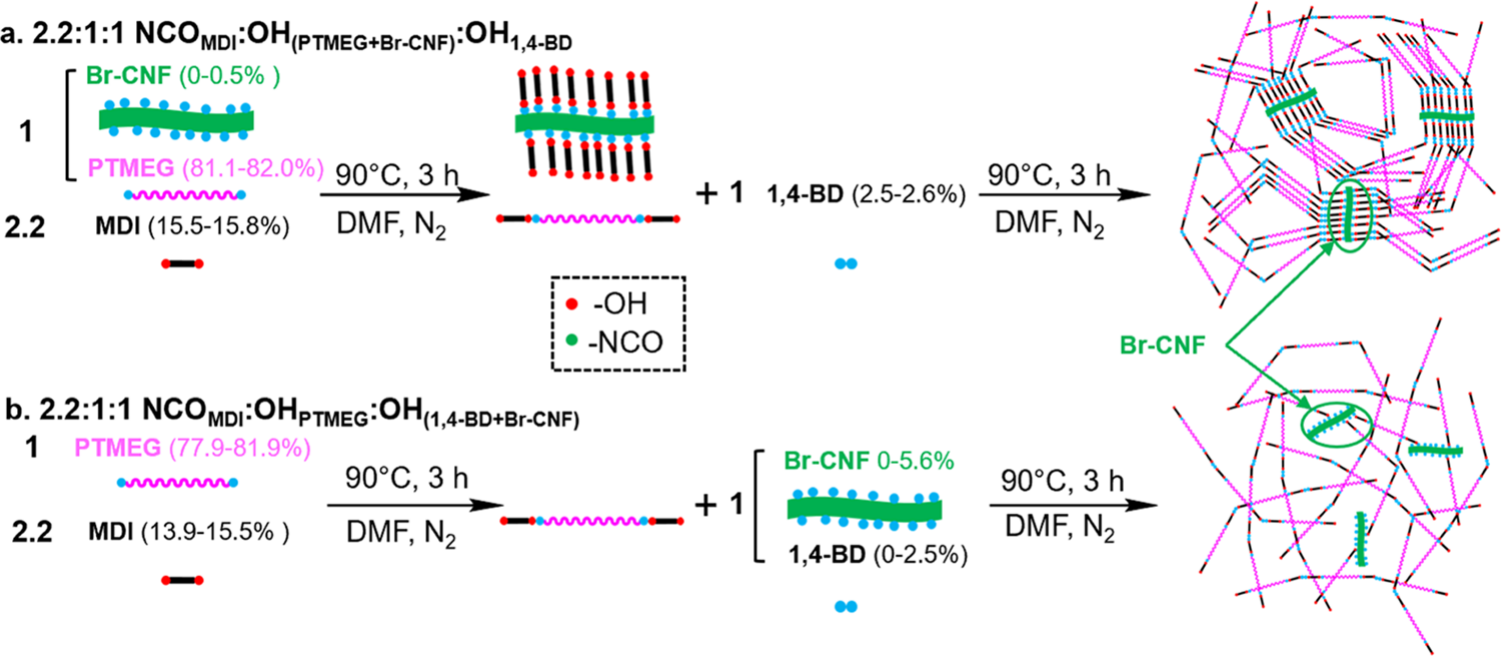
Scheme for Polyurethane Synthesis with Br-CNFs Serving as (a) a Chain Extender or (b) a Polyol at the Same 2.2:1:1 NCO_MDI_/OH_PTMEG+Br-CNF_/OH_1,4-BD+Br-CNF_ Molar Ratio

To prepare PU/CNF composites using Br-CNF as part of the extender at a fixed 2.2:1:1 NCO_MDI_/OH_PTMEG_/OH_1,4-BD+Br-CNF_ molar ratio (Scheme 1a), PTMEG (*M*_n_: 2900 Da, 1.72 mmol of OHs, 2.5 g) was reacted with MDI (3.80 mmol of NCOs, 0.47 g) in 15 mL of DMF, degassed (Branson 2510) for 1 min, purged with N_2_ for 10 min, and then sealed and heated to 90 °C with stirring for 3 h to form a prepolymer. Br-CNF (0.5% w/v in DMF) was added at 1.8 or 5.4 wt%, i.e., 11 or 35 mL (0.19 and 0.61 mmol of available OHs), to predissolve 1,4-BD (0.069 and 0.051 g, 1.54 and 1.12 mmol of OHs), degassed for 1 min, then added to the prepolymer under constant stirring at 90 °C for another 3 h, and finally quenched in an ice bath to stop the reaction. Without any 1,4-BD, PU/CNF with Br-CNF (0.5 w/v%, 0.61 mmol of available OHs, 35 mL) as the lone extender was also synthesized at a fixed 2.2:1:0.35 NCO_MDI_/OH_PTMEG_/OH_Br-CNF_ molar ratio for comparison.

### Br-CNF as a Polyol

The PU/CNF composites with Br-CNF serving as a polyol (Scheme 1b) were prepared at varying Br-CNF contents (0–0.5 wt%) at a fixed 2.2:1:1 NCO_MDI_/OH_PTMEG+Br-CNF_/OH_1,4-BD_ ratio. Br-CNF dispersions were diluted with DMF to 20 mL in 0.0075, 0.015, 0.03, 0.045, and 0.075 w/v%, degassed (Branson 2510) for 1 min, and purged N_2_ for 10 min. PTMEG (*M*_n_: 2900 Da, 1.72 mmol of OHs, 2.5 g) and MDI (3.8–3.92 mmol of NCOs, 0.47–0.49 g) were added to each dispersion in a high-vacuum silicone-grease-sealed 50 mL round bottom flask in a 90 °C oil bath under stirring to react for 3 h to form a prepolymer. 1,4-BD (0.86 mmol, 0.078 g) was added to react for another 3 h and then quenched to end the reaction. Reactions were repeated with 1.8 mL of Br-CNF (0.5 w/v%) at 2.1:1:1 and 2:1:1 NCO_MDI_/OH_PTMEG+Br-CNF_/OH_1,4-BD_ molar ratios to investigate excess MDI effects. Lower molecular weight PTMEG (*M*_n_: 1000 Da) was used with 1.8 mL of Br-CNF (0.5 w/v%) at a 2.2:1:1 NCO_MDI_/OH_PTMEG+Br-CNF_/OH_1,4-BD_ molar ratio. Films were cast from various viscous reaction mixtures of various PU/CNF compositions as well as Br-CNF alone in glass Petri dishes and dried in an oven at 60 °C for 2 days. The volumes of circular films were calculated from the thickness and diameter measured by micrometers and graduated scales to estimate densities.

### Characterization of the PU/CNF Film

The morphology of the PU/CNF film was imaged by optical microscopy (Leica DM2500) in transmission mode and under cross-polar mode. For attenuated total reflectance (ATR) Fourier transform infrared spectroscopy, each PU film was scanned by a Thermo Nicolet 6700 spectrometer under ambient conditions from an accumulation of 128 scans at a 4 cm^–1^ resolution from 4000 to 400 cm^–1^. To determine glass transition (*T*_g_) and melting (*T*_m_) temperatures of the PU/CNF film, each ca. 10 mg of sample was cooled by liquid nitrogen to −100 °C and scanned at 10 °C/min to 50 °C by differential scanning calorimetry (DSC, DSC-60 Shimadzu). The tensile properties of films (40 × 14 × 0.4 mm^3^) were measured using an Instron 5566 tensile tester with a static 5 kN load cell, ca. 20 mm gauge length, and 20 mm/min crosshead speed to break and to 400% strain in cyclic mode. For each data point, at least three films were tested with the average value and standard deviation reported. The modulus was determined by the initial slope of the strain–stress curve. Engineering stress (σ) was calculated from *F*/*A*_0_, where *F* is the applied load (N) and *A*_0_ is the initial cross-sectional area (m^3^). Engineering strain (ε) was calculated by Δ*L*/*L*_0_, where Δ*L* is the extension (mm) of the sample and *L*_0_ is the initial sample gauge length (mm).

## Results and Discussion

### Characteristics of Br-CNFs

Br-CNFs were optimally synthesized by one-pot 2-bromopropionyl esterification of rice straw cellulose (5:1 BPB to AGU molar ratio, 23 °C, 6 h) and in situ ultrasonication (50% amplitude, 30 min) in DMF^63^ to be ribbon-like with 4.6 ± 1.8 nm thickness (*T*), 29.3 ± 9.2 nm width (*W*), and ca. 1 μm length (*L*) (Figure S1a,b). Br-CNF geometries are uniquely anisotropic, showing over 6 *W*/*T* and 213 *L*/*T* ratios. 2-Bromopropionyl esterification converts the OHs in the less ordered region of cellulose to 2-bromopropionyl esters to endow them with organic compatibility and to facilitate the direct disintegration by ultrasonication of 2-bromopropionyl esterified cellulose into homogeneously dispersed Br-CNFs, all in the same organic medium DMF. The level of substitution (ρ) quantified by ^1^H NMR (Figure S2) was 0.48, showing that nearly half of the surface OHs were converted to 2-bromopropionyl esters. The remaining 52 % surface OHs, equivalent to 3.5 mmol of OHs/g Br-CNF by eq 2, remained available to react with MDI (Table 2). The XRD of Br-CNFs displayed 2θ peaks at 14.6, 16.5, and 22.5 °, corresponding to the respective (11̅0), (110), and (200) monoclinic Iβ lattice planes of cellulose, respectively (Figure S3); 0.48 CrI of Br-CNF showed the retention of 69 % crystallinity of the original cellulose (CrI: 0.69).

**Table 2:** CNF Characteristics: Dimensions, Crystallinity, Br Content/Degree of Substitution, and Available OH Content

thickness (nm)	width (nm)	length (nm)	crystallite dimension (nm)	Crl	Br content (mmol/g)	level of substitution	OHs content (mmol/g)
4.6	29.3	ca. 1000	1.45	0.48	3.2	0.48	3.5

Br-CNFs are similar in thickness (*T* = 4.6 nm) to highly hydrophobic ODE-CNF (*T* = 4.4 nm, *W* = 4.1 nm, *L* = 1.7 μm),^66^ both are thicker than hydrophilic TEMPO-CNF (*T* = 1.5 nm, *W* = 2.1 nm, up to 1 μm long),^13^ all ca. 1 μm or longer and derived from the same rice straw cellulose. Br-CNF (*W* = 29.3 nm) is, however, considerably wider than ODE-CNF and TEMPO-CNF, i.e., by 7 and 14 times, respectively. A *W*/*T* ratio of 6 of the cross section gives Br-CNF highly anisotropic lateral dimensions than the near isotropic *W*/*T* ratios of ODE-CNF and TEMPO-CNF; the latter two disintegrated by high-speed blending in water. These lateral dimensional and aspect ratio differences indicated the specific ultrasonication applied to be less intensive to disintegrate 2-bromopropionyl esterified cellulose in the less ordered domains into CNFs compared to the aqueous high-speed blending of either hydrophobic ODE-cellulose or hydrophilic TEMPO-cellulose. Br-CNFs (CrI: 0.48; Figure S3) are slightly less crystalline than ODE-CNF (CrI: 0.52)^66^ but clearly less crystalline than TEMPO-CNF (CrI: 0.63).^13^ The reduced crystallinity of Br-CNF was attributed mainly to the chemical reaction of cellulose, i.e., 2-bromopropionyl esterification reduced the crystallinity of cellulose (CrI: 0.69) to the Br-cell (CrI: 0.50)^63^ to signify the more robust 2-bromopropionyl esterification in DMF in comparison to lesser effects on crystallinity from the less intensive telomerization^66^ or TEMPO-oxidation.^13^ The significantly retained crystallinity (CrI: 0.48) and largely available surface OHs (3.5 mmol/g) made Br-CNF uniquely surface-reactive polyols with a crystalline core as potential covalent bonded reinforcement in TPU synthesis.

### Br-CNF as a Chain Extender in PU Synthesis at 2.2:1:1 NCO/OH/OH

**Figure 1:**
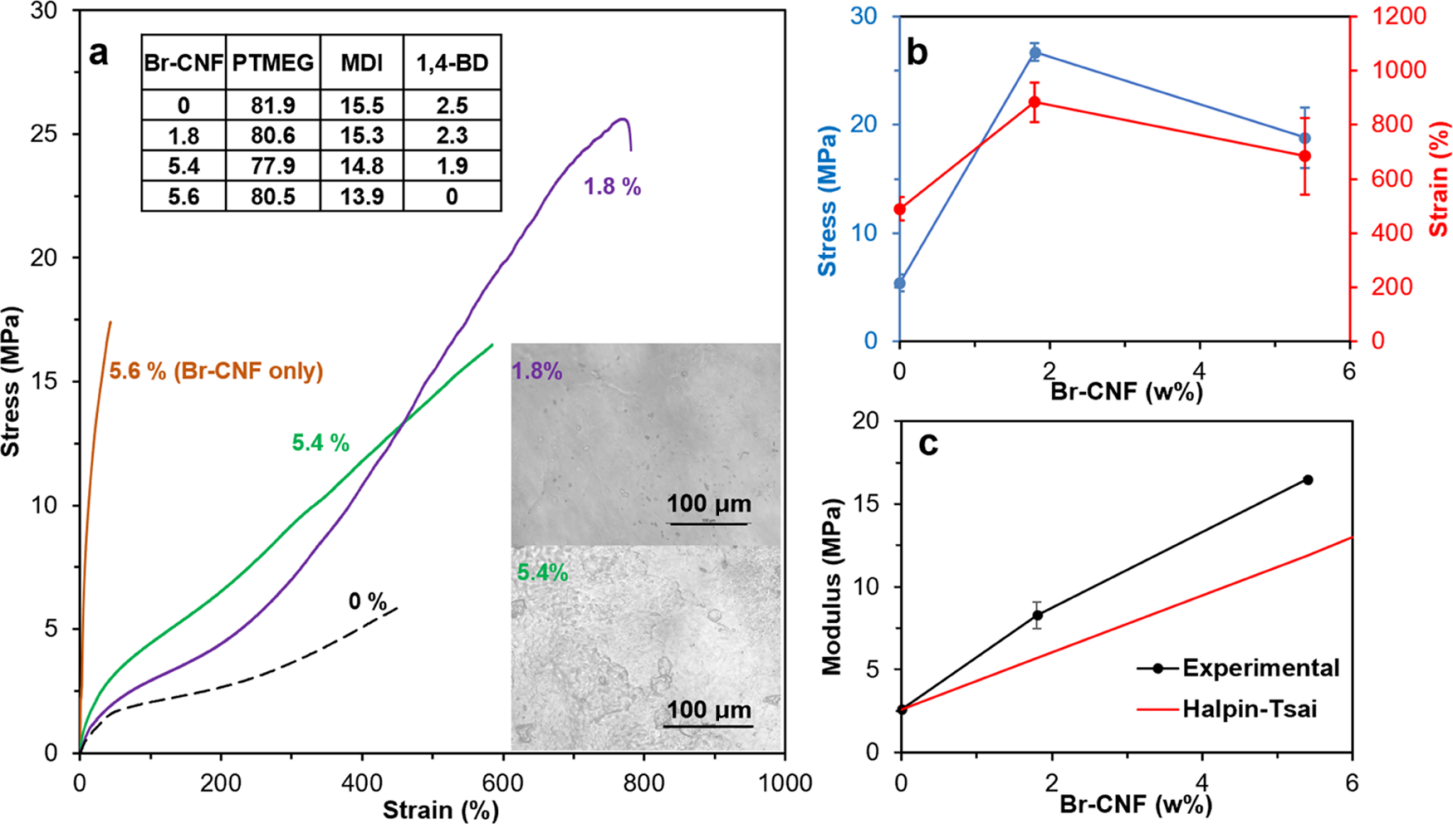
Tensile properties of PU synthesized with Br**-**CNF as a chain extender to replace 11 and 30 mol% 1,4-BD hydroxyls at 2.2:1:1 NCO_MDI_/OH_PTMEG_/OH_1,4-BD+Br-CNF_: (a) representative stress–strain curves for PU/CNF films (*n* ≥ 3) with Br-CNF (wt) compositions and photographic images of PU/CNF films; (b) tensile stress and strain-to-failure; and (c) experimental and Halpin–Tsai simulated elastic moduli (MPa) (*n* ≥ 3).

Br-CNF was incorporated as a chain extender to partially replace 11 and 35 mol% OH in 1,4-BD or 1.8 and 5.4 wt% Br-CNF in PU syntheses at a fixed 2.2:1:1 NCO_MDI_/OH_PTMEG_/OH_1,4-BD+Br-CNF_ molar ratio (Scheme 1a and Figure 1). Br-CNF was also incorporated as the only chain extender at 5.6 wt%, equivalent to 35 mol % OHs of 1,4-BD, for comparison. Upon replacing 11 mol% OHs of 1,4-BD with 1.8 wt% Br-CNF (Figure 1b,c), the elastic modulus, tensile strength, and strain-to-failure significantly increased from 2.6 to 8.3 MPa, 5.4 to 26.7 MPa, and 490 to 883 %, respectively. Replacing over 3 times of 1,4-BD OHs (35 mol%) with Br-CNF (5.4 wt%) further doubled the modulus to 16.5 MPa but lowered the strength by 30 % to 18.8 MPa and strain by 22 % to 684 %. The enhanced elastic modulus and tensile stress were attributed to linking the MDI-capped soft segments with multiple OHs of Br-CNF like cross-linkers, instead of the short 4-C extender. The increased strain-to-failure was attributed to the strengthened soft domains from hydrogen bonding between the remaining unreacted OH on Br-CNF and PTMEG. This modulus increase essentially linearly with Br-CNF content further signifies the contribution of multiple cross-linked Br-CNF with MDI-capped soft segments. The more heterogeneous appearance of films with 5.4 wt % Br-CNF suggests possible agglomeration and/or phase separation of Br-CNF to lead to lowered tensile stress and strain-to-failure (Figure 1a). Furthermore, using 5.6 wt% Br-CNF (equivalent to 35 mol% OHs of 1,4-BD) as the only extender, the modulus drastically increased from 16.5 to 173 MPa, while the strain significantly reduced from 684 to 46 %, but tensile stress only slightly decreased from 18.8 to 17.0 MPa. As the lone chain extender, 5.6 wt% Br-CNF only provided 35 mol% OHs of 1,4-BD, insufficient to link all of the MDI-capped soft segments to turn the elastomeric PU to high-modulus plastic.

The Halpin–Tsai model that predicts the modulus of short fiber reinforced composites with a perfectly aligned, homogeneously mixed, and constant fiber volume fraction in a continuous matrix^67^ was used to compare with the experimental values. The predicted modulus from the Halpin–Tsai model is expressed as follows
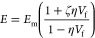
3
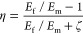
4where *E* is the longitudinal modulus of the unidirectional composite, *V*_f_ is the fiber volume fraction based on the mass fraction of Br-CNF in PU with their respective estimated densities of 1.2 and 1.1 g/cm^3^, *E*_m_ and *E*_f_ are the respective matrix and fiber moduli, and ζ is a shape factor for Br-CNF and defined^68^ as
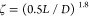
5where *L* is the 1 μm length of Br-CNF and *D* is the diameter or the geometrical mean (11.7 nm) of Br-CNF thickness (4.6 nm) and width (29.3 nm).

From the Halpin–Tsai model simulation,^67,68^ elastic moduli for PU with 1.8 and 5.4% Br-CNF were 5.7 and 11.9 MPa, respectively, ca. 30 % lower than the respective experimental values of 8.3 and 16.5 MPa. The higher experimental modulus than that predicted by the Halpin–Tsai model supports the presence of new covalent bonding between Br-CNF and MDI when applied as an extender. As a chain extender, the optimal Br-CNF content was 1.8 wt% to significantly improve all three tensile properties, i.e., an over 3 times increase in modulus, a nearly 4 times increase in strength, and an 80% increase in strain. Similarly, the overall toughness also reached its highest at 1.8 wt%.

### Br-CNF as a Polyol in PU Synthesis

#### Br-CNF Loading

**Figure 2:**
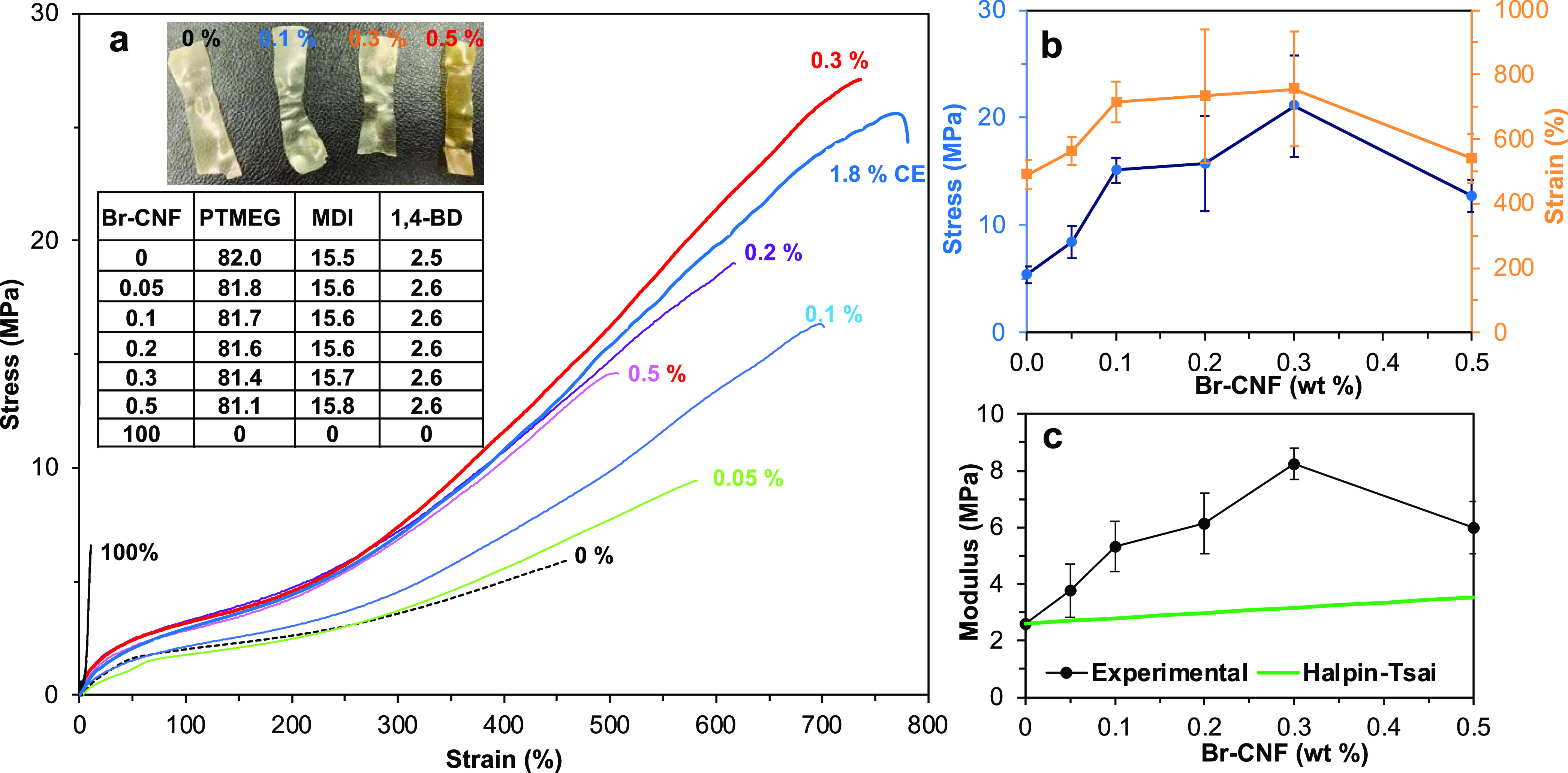
Tensile properties of PU synthesized with Br-CNFs as polyols replacing 0.3, 0.6, 1.2, 1.8, and 3.1 mol% OHs in PTMEG (*M*_n_: 2900 Da) diol at a 2.2:1:1 NCO_MDI_/OH_PTMEG+Br-CNF_/OH_1,4-BD_ mole ratio: (a) representative stress–strain curves for PU/CNF films with 0.05, 0.1, 0.3, and 0.5 wt% Br-CNF; photographic images of the upper portions of fractured samples and 1.8 wt% Br-CNF (11 mol % of OH in 1,4-BD) as a chain extender (CE) from Figure 1a for comparison; (b) tensile stress (MPa) and strain-to-failure (%); and (c) experimental and Halpin–Tsai simulated elastic moduli (MPa) of PU/CNF films (*n* ≥ 3).

Br-CNF with 3.5 mmol of OHs/g was also used as a polyol to replace 0.3, 0.6, 1.2, 1.8, and 3.1 mol% OHs of PTMEG diols to synthesize a prepolymer with 10 mol% excess of MDI, i.e., a 2.2:1:1 NCO_MDI_/OH_PTMEG+Br-CNF_/OH_1,4-BD_ mole ratio, to ensure capping all Br-CNF surface OHs. This partial replacement of diol with the Br-CNF polyol represents 0.05, 0.1, 0.2, 0.3, and 0.5 wt% Br-CNF in the PU/CNF composites. The colorless PU turned yellowish with increasing Br-CNF content and into golden color with 0.5 wt% Br-CNF (Figure 2a). With up to 0.3 wt% Br-CNF, the elastic modulus increased by over 3 times from 2.6 to 8.3 MPa and the tensile strength by nearly 4 times from 5.4 to 21.1 MPa, while the strain also increased by 54 % from 490 to 755 % (Figure 2b,c). However, with further increased Br-CNF content to 0.5 wt%, tensile modulus, strength, and strain reduced to 6.0 MPa, 15.7 MPa, and 539%, respectively, i.e., levels near or below those with 0.1 wt% Br-CNF. The increases in all three mechanical properties were attributed to improved dispersion due to 2-bromopropionyl ester-functionalized Br-CNF surfaces and covalent bonding between Br-CNF surface OHs and MDI. The outstanding reinforcement effects on the modulus and tensile strength were attributed to the crystalline core of Br-CNF and surface OHs covalently bonded with MDI, serving as additional and new kinds of hard segments. Meanwhile replacing diol with the Br-CNF polyol in the soft segments could also enhance hydrogen-bonding interactions between unreacted Br-CNF surface OHs and PTMEG to increase stretchability. In addition, the ability of Br-CNF to realign along the loading direction may be another reason for increased tensile strength and stretchability. That much reduced tensile stress, strain-to-failure, and elastic modulus with 0.5 wt% Br-CNF may be due to inter Br-CNF association, reducing their reaction with MDI and hydrogen bonding with PTMEG. Comparably, all experimental elastic modulus values of PU with Br-CNF as a polyol were over 2 times or higher than the Halpin–Tsai model simulated values (Figure 2c), indicating the extreme effectiveness of strong covalent bonding between Br-CNF and MDI. This observation illustrated that surface OHs on Br-CNF are more reactive to free MDI as a polyol but less accessible to the MDI-capped PTMEG prepolymer as a chain extender. In fact, the same modulus of 8.3 MPa was achieved with Br-CNF serving as either a polyol or a chain extender, but requiring only one-sixth in the polyol role (0.3 wt%) of that in the chain extender role (1.8 wt%). The optimal molar replacement of Br-CNF hydroxyls for those in the PTMEG soft segment or 1,4-BD chain extenders was 1.8 or 11 mol%, respectively.

These findings show, for the first time, that a mere 0.3 wt% Br-CNF quantity can significantly enhance the tensile modulus (3.2×) and strength (3.9×) while also improving the strain-to-failure (1.5×). In all prior work involving cellulose or nanocellulose either as a filler^21−24,27,28^ or in PU synthesis,^29−34^ improvement in all three tensile properties was only reported in three cases, i.e., 5 wt% MCC,^29^ 1 wt% MFC,^30^ and sonication-assisted 1 wt% CNC,^32^ all with the shorter PTMEG (*M*_n_: 1000 Da; Table 1), while the PUs synthesized with the commonly used shorter PTMEG are expected to have higher modulus but reduced strain, and the MDI quantities were also double. Among them, only two documented Fourier transform infrared (FTIR) evidence of new covalent bonding formation between MFC^30^ or CNC^32^ and isocyanates. Furthermore, Br-CNF is homogeneously dispersed in DMF without any pretreatment nor shear force mixing, a stark contrast to the extra and necessary processes of freeze-drying,^32^ solvent exchange^33,34^ and then aided by homogenization,^31^ or sonication^32−34^ to disperse hydrophilic nanocelluloses. Uniquely, Br-CNF is not only efficiently synthesized, i.e., one-pot esterification and in situ disintegration, directly from cellulose but also robust in reactivity to serve dual roles as either a polyol or a chain extender in the synthesis of PU. Most significantly, the quantity of the toxic MDI was significantly reduced to half.

#### MDI Optimization

**Figure 3:**
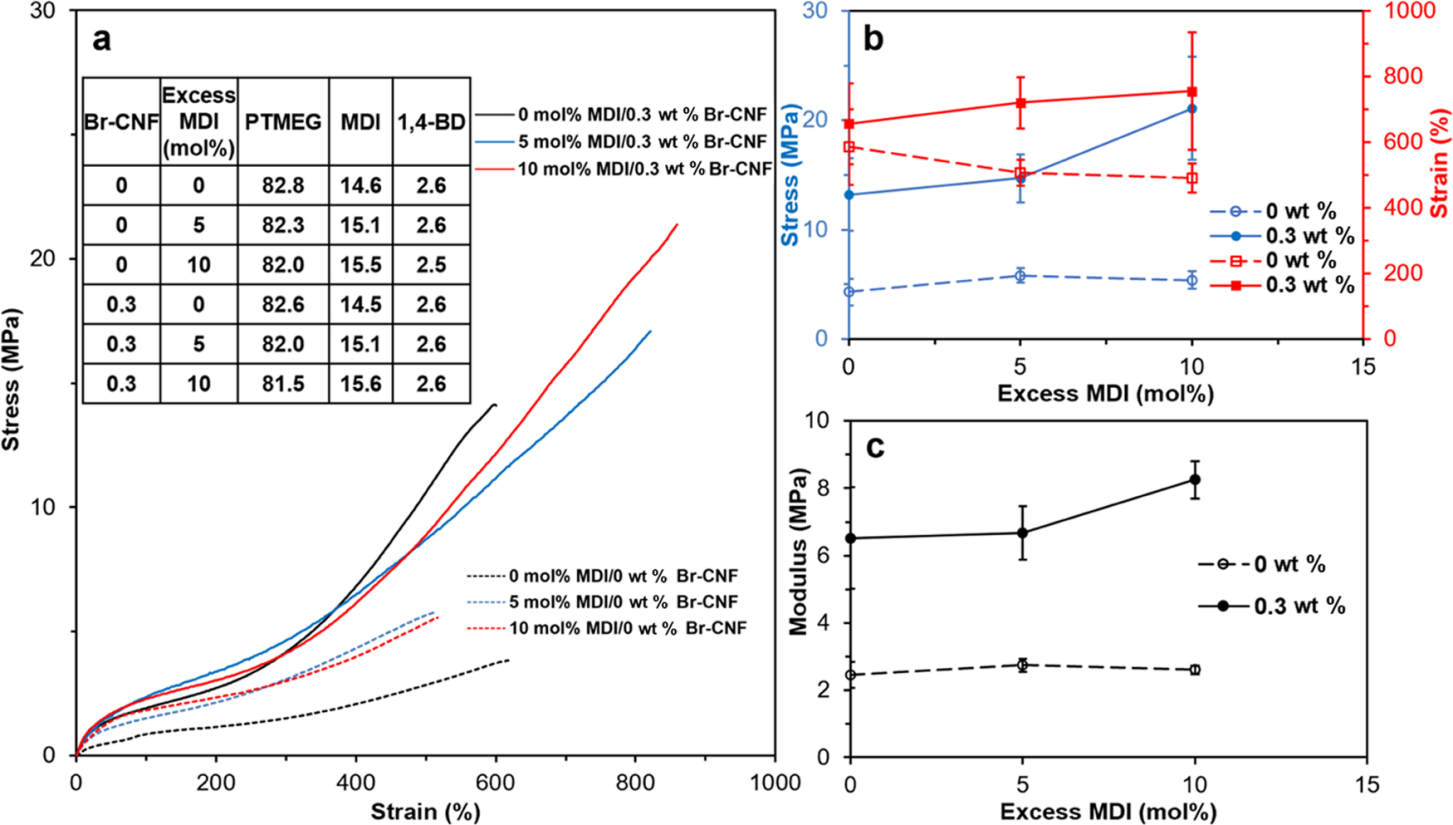
Tensile properties of PU synthesized with 0.3 wt % Br-CNF polyol in PTMEG (*M*_n_: 2900 Da) diol with 0–10 mol% excess MDI (2–2.2:1:1 NCO_MDI_/OH_PTMEG+Br-CNF_/OH_1,4-BD_): (a) representative stress–strain curves with compositions in wt %; (b) tensile stress and strain; and (c) modulus (*n* ≥ 3).

In the attempt to further reduce the diisocyanate quantity, the molar excess MDI was reduced from 10 to 0 mol% in the synthesis of a prepolymer with 0.3 wt% Br-CNF as a polyol replacing 1.8 mol% PTMEG hydroxyls (Figure 3a). Generally, both modulus and tensile strength displayed a positive correlation to excess MDI mol %; meanwhile, a negative correlation was observed for strain-to-failure (Figure 3b,c). In the absence of Br-CNF, both elastic modulus and tensile stress of the PU control slightly increased from 2.5 to 2.8 MPa and 4.3 to 5.8 MPa, respectively, whereas strain-to-failure slightly decreased from 585 to 507 % with 5 mol% excess MDI, but showing no further change with 10 mol% excess MDI, indicating 5 mol % excess MDI to be adequate to cap PTMEG diols in the synthesis of PU. With 0.3 wt% Br-CNF, all three tensile modulus, stress, and strain-to-failure moderately increased from 6.5 to 8.3 MPa, 13.2 to 21.1 MPa, and 656 to 755 %, respectively, with the increase of excess MDI to 10 mol%. More excess MDI required with Br-CNF than the control suggested that surface OHs on Br-CNF may be less accessible than those of the PTMEG diol. It is also possible that surface Br esters determined by ^1^H NMR may be overestimated due to the DMF-to-acetone solvent exchange preparation that left out some less substituted Br-cellulose, leading to underestimated OHs content of Br-CNF. Further increasing Br-CNF content from 0.3 to 1 wt% (Figure S4) w/o excess MDI moderately increased strength (1.2×) and strain-to-failure (1.2×) but sacrificed 20% elastic modulus. The optimal Br-CNF as a polyol w/o excess MDI was determined to be 1 wt %, nearly 3 times higher than optimal 0.3 wt% Br-CNF content at 10 mol% excess MDI. Thus, 10 mol% excess MDI not only further enhances the tensile properties of PU/CNF composite but also requires significantly less Br-CNF. Unlike prior PU synthesized with a constant 2:1 NCO_MDI_/OH_PTMEG_^29,30,32,34^ ratio without consideration of cellulose OHs, this work rationally targets the OHs of both the Br-CNF polyol and the PTMEG diol stoichiometrically to isocyanate to significantly improve all tensile properties of PU, i.e., 3.2× modulus, 3.9× strength, and 1.5× strain-to-failure, with mere 0.3 wt % Br-CNF as a polyol and 10 mol% excess MDI.

#### PTMEG Chain Length

**Figure 4:**
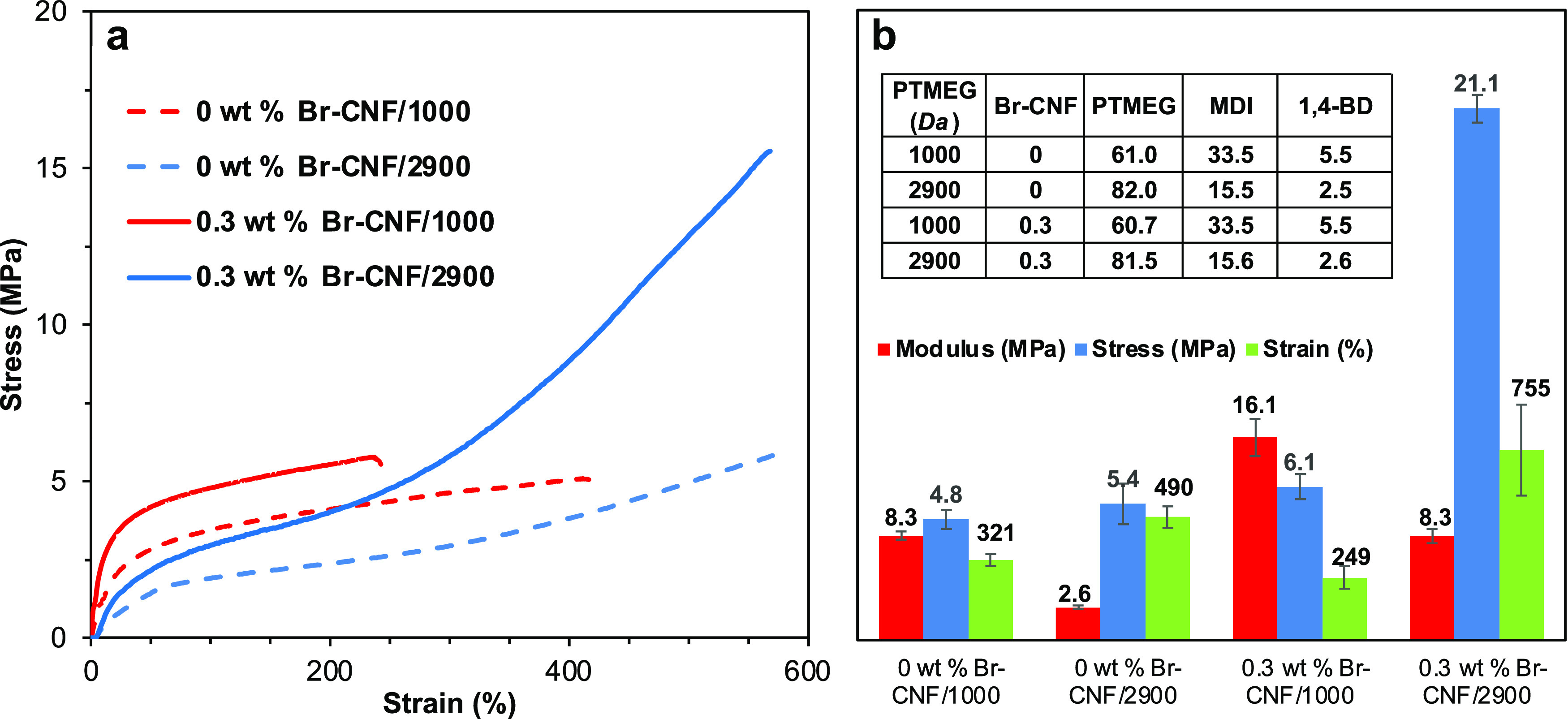
Tensile properties of PU synthesized with Br-CNFs as polyols and either 1000 or 2900 Da PTMEG at consistent 2.2:1:1 NCO_MDI_/OH_PTMEG+Br-CNF_/OH_1,4-BD_: (a) representative stress–strain curves and (b) elastic modulus, tensile stress, and strain-to-failure (*n* ≥ 3), with compositions (wt %).

While using longer PTMEG (*M*_n_: 2900 Da) than the commonly reported shorter PTMEG (*M*_n_: 1000 Da) has the advantage of reducing MDI usage, the lower elastic modulus and higher strain of PU are expected and were confirmed by the control PU synthesized without Br-CNF (Figure 4a). With the optimal 0.3 wt% Br-CNF as a polyol and 10 mol% excess MDI, the elastic modulus nearly doubled from 8.3 to 16.1 MPa, and strength and strain modestly increased and decreased, respectively, for the shorter PTMEG, while in contrast, the modulus increased from 2.6 to 8.3 MPa by over 3 times, stress nearly quadrupled from 5.4 to 21.1 MPa, and strain nearly doubled from 490 to 755% with the nearly 3 times longer PTMEG (Figure 4b). Therefore, while incorporating 0.3 wt% Br-CNF as a polyol most significantly improved the modulus of PU with shorter PTMEG, the effect on PU with the longer PTMEG was significant in all three tensile properties. The more significant reinforcement effects of Br-CNF on PU synthesized with longer PTMEG (*M*_n_: 2900 Da) support the notion that both covalent bonding between Br-CNF and MDI and hydrogen bonding between B-CNF and PTMEG were maximized to give the best mechanical performance. Also, adding 0.3 wt% Br-CNF as a polyol would minimize the negative effects of the longer PTMEG on the modulus to retain the same modulus (8.3 MPa) as short PTMEG (*M*_n_: 1000 Da).

While the optimal 1.8 wt% Br-CNF as an extender led to 27 % higher tensile strength (26.7 vs 21.1 MPa), 17 % higher strain-to-failure (883 vs 755 %), and the same elastic modulus (8.3 MPa) compared to the values obtained when it played the role of a polyol, the slight strength enhancement with 6 times Br-CNF is indicative of the more efficient covalent bonding of Br-CNF with MDI as a polyol than that as a chain extender. With similar PTMEG, MDI, and 1,4-BD contents, 0.3 wt% Br-CNF as a polyol capped by 10 mol% excess MDI with PTMEG (*M*_n_: 2900 Da) was optimal to produce the most significantly reinforced PU film with 3.2× modulus, 3.9× strength, and 1.5× strain-to-failure while reducing MDI usage to 15.7 wt%.

Lastly, ethylene glycol (EG) was used to replace 1,3-BD to improve diffusion and stiffen the diol-MDI-diol hard segments for potential further strength enhancement. In addition, Br-CNF was used to replace both PTMEG and EG chain extender OHs at optimal 1.8 and 11 mol%, respectively. The tensile strength of PU with Br-CNF in both roles did not produce any synergistic or even additive effect. (Figure S5). At a total of 2.1 % mass content, Br-CNF may have agglomerated to heterogeneously phase separate to impede their covalent bonding with MDI and/or hydrogen bonding with each other. Therefore, the optimal reinforcing effect of Br-CNF requires a balance of achieving maximal covalent bonding to MDI as well as maximal hydrogen bonding with PTMEG.

#### ATR and DSC Spectra of PU/CNF Composites

**Figure 5:**
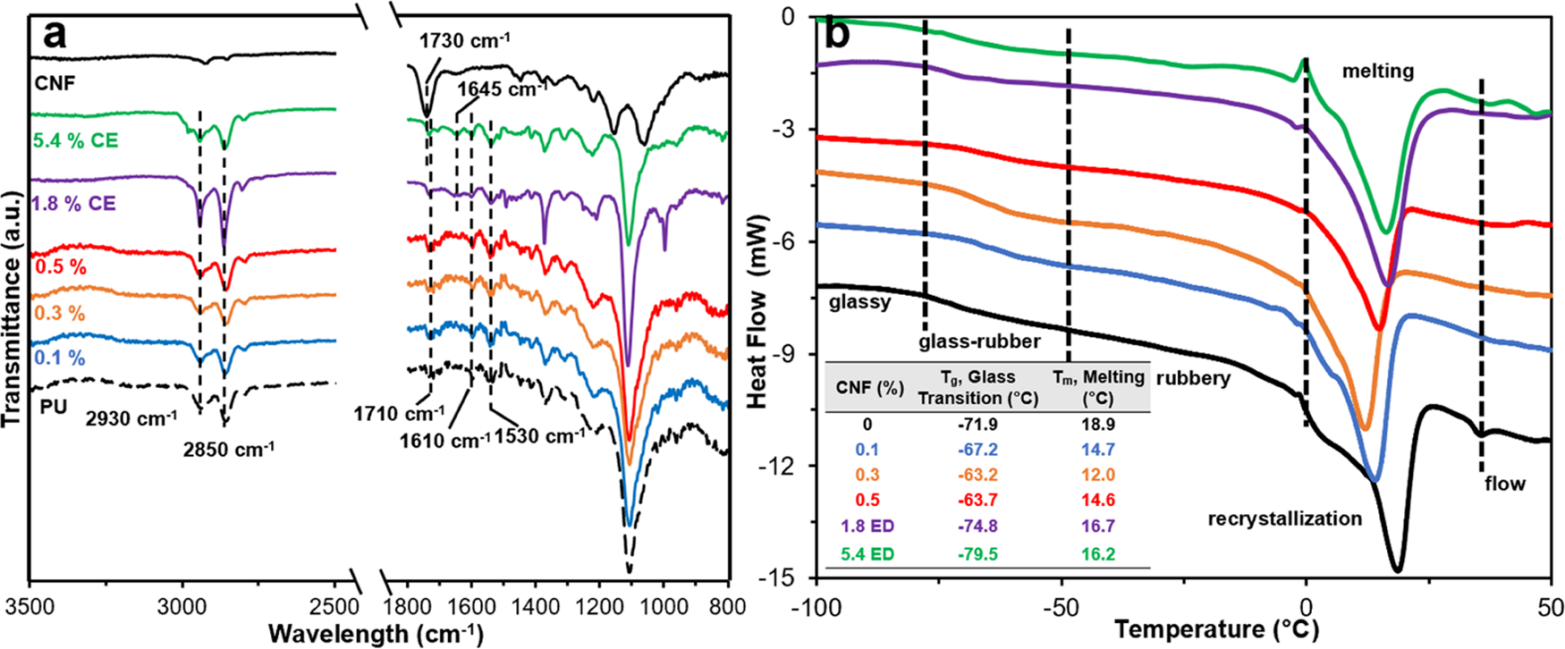
Characteristics of PU/CNF composites with Br-CNF as a polyol (0.1, 0.3, 0.5 wt %) or an extender (1.8 and 5.4 wt%): (a) attenuated total reflectance (ATR) spectra and (b) differential scanning calorimetry thermograms.

The presence of the urethane link in PU and PU/CNF composite films was clearly evident in their ATR spectra (Figure 5a), showing C=O peaks at 1709 cm^–1^ (hydrogen bonded) and 1730 cm^–1^ (free), the C–N asymmetric stretching peak at 1610 cm^–1^, and the N–H bending peak at 1530 cm^–1^. For films with 1.8 and 5.4 wt% Br-CNF as chain extenders, the detection of a new carbonyl peak at 1645 cm^–1^ gave evidence of the reaction between the Br-CNF surface OHs and MDI. In those with Br-CNF as a polyol, however, no new peak was observed due to the extremely low quantities up to 0.5 wt%. The effects of Br-CNF from covalent bonding with MDI, either as a polyol or a chain extender, as well as their interactions with the soft segments, were further elucidated by their thermal behaviors (Figure 5b). Glass transition temperature *T*_g_ increased from −71.9 to −63.7 °C with increasing 0–0.5 wt% Br-CNF polyol but decreased to −79.5 °C with increasing 0–5.4 wt% Br-CNF extender. The endothermic recrystallization peak for MDI-1,4-BD-MDI hard domains remained constant at −1.5 °C and independent of Br-CNF contents and roles, indicative of no Br-CNF effects on the original PU hard domain size and distribution. As a polyol, the effective reaction between Br-CNF and MDI introduces new Br-CNF-MDI carrying polyisocyanate terminals among the diols, also bearing isocyanate terminals, to reduce the segmental motion of the soft segments to decrease *T*_g_. As an extender, the higher Br-CNF contents and the availability of more unreacted OHs on the Br-CNF surface would hydrogen bond with PTMEG (−OR), suppressing PTMEG phase separation into smaller soft domains, thus lowering *T*_g_. Melting temperature *T*_m_ decreased from 18.9 to 12.0 °C with increasing Br-CNF contents from 0 to 0.3 wt% as a polyol, while decreased slightly to 16.2 °C with Br-CNF as extender up to 5.4 wt %. In either polyol or extender role, increasing Br-CNF contents is expected to increase the extent of covalent bonding to MDI, lowering the extent of MDI-1,4-BD-MDI hard domains to lower *T*_m_. The stronger covalent bonding between Br-CNF and MDI may be the reason for higher *T*_m_ reduction with Br-CNF as a polyol than that as an extender. One possible explanation for elevated *T*_m_ (14.6 °C) with increased Br-CNF as a polyol from 0.3 to 0.5 wt% may be that Br-CNFs extensively covalent bonded with MDI behave as cross-linkers and new hard domains to suppress mobility of the original MDI-1,4-BD-MDI hard domains.

#### Cyclic Tensile Properties of the Polyurethane Film with Br-CNF as a Polyol

**Figure 6:**
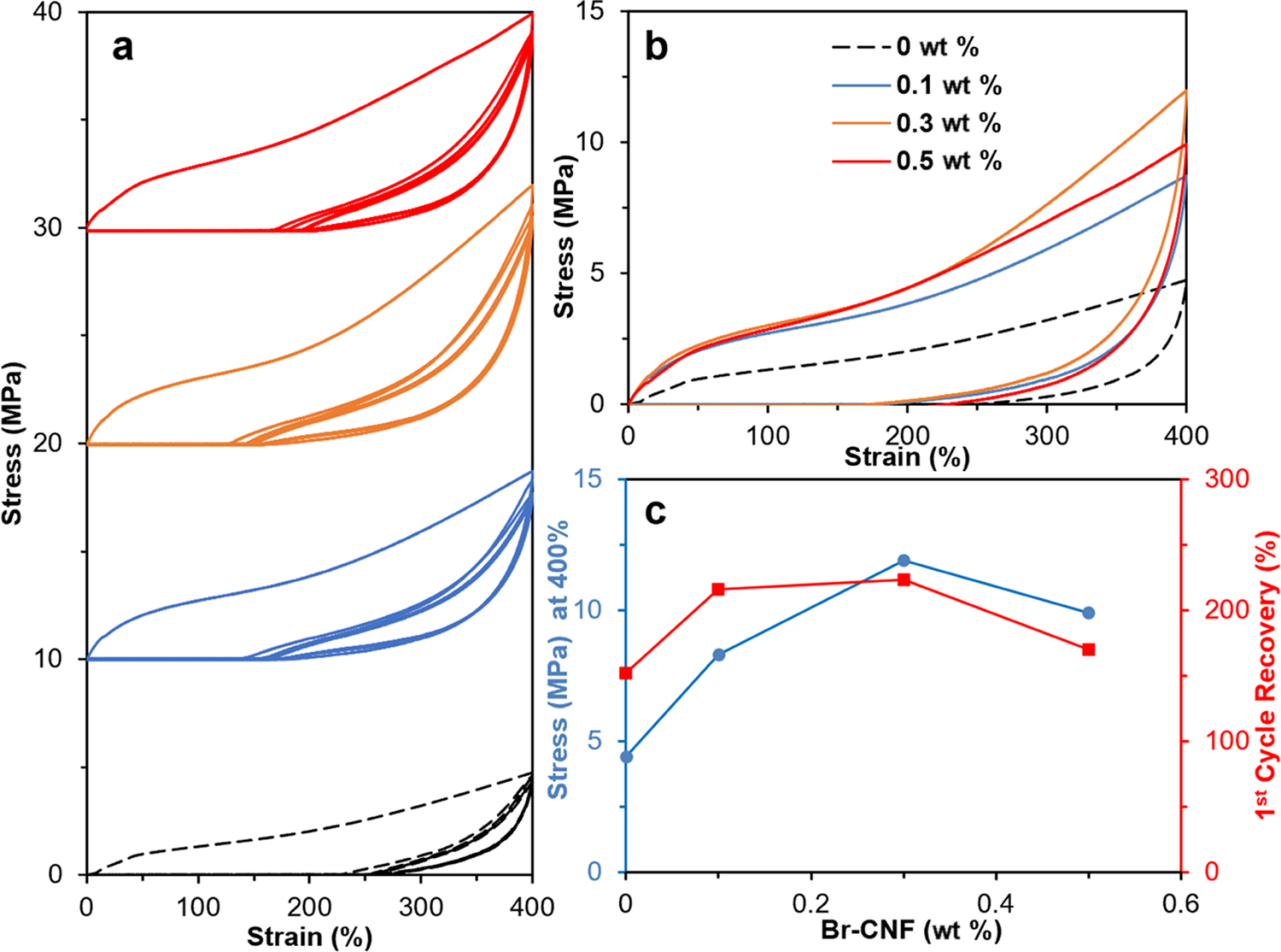
Cyclic tensile properties of PU/CNF films with Br-CNF (wt %) as a polyol at 400% strain for five cycles: (a) cyclic stress–strain curves; (b) first cyclic stress–strain curves, and (c) first tensile stress and cycle recovery (%). All films are synthesized with at 2.2:1:1 NCO_MDI_/OH_PTMEG+Br-CNF_/OH_1,4-BD_ and PTMEG (*M*_n_ = 2900 Da).

To further investigate elastic and inelastic behaviors, uniaxial cyclic tensile strain/stress curves for PU/CNF films with 0, 0.1, 0.3, and 0.5 wt% Br-CNF (0, 0.6, 1.8, and 3.1 mol% PTMEG OHs) as polyols were evaluated at up to 400 % strain (Figure 6a). The first cycle tensile stress significantly increased from 4.4 to 11.9 MPa with increasing Br-CNF contents to 0.3 wt%, and then lowered to 9.9 MPa at 0.5 wt %. In the first cycle, the strain recovery for PU was 152 % and increased to 223 % with 0.3 wt% Br-CNF polyol (Figure 6b,c). The behavior of decreasing stress at 400% with an increasing number of cycles, or the stress relaxation phenomenon, was observed for all three PU/CNF composites. At the end of the fifth cycle, stress at 400 % strain decreased from 8.3 to 7.0 MPa, 11.9 to 9.5 MPa, and 9.9 to 8.5 MPa, corresponding to 0.1, 0.3, and 0.5 wt% Br-CNF contents, respectively, in contrast to the lacking stress relaxation for PU control. Those observed stress relaxation phenomena possibly caused by realignment of Br-CNF along the loading direction indicated the existence of irreversibility with Br-CNF as a polyol at a high strain of 400 %. Nevertheless, the stress after five cycles of PU with Br-CNF polyols was significantly higher than that of PU alone. Both the highest 11.9 MPa tensile stress and 223 % first cycle recovery observed at 0.3 wt% Br-CNF as a polyol confirmed this to be the optimal PU/CNF composition to generate the most resilient film.

#### Orientation of Br-CNF in PU along the Loading Direction

**Figure 7:**
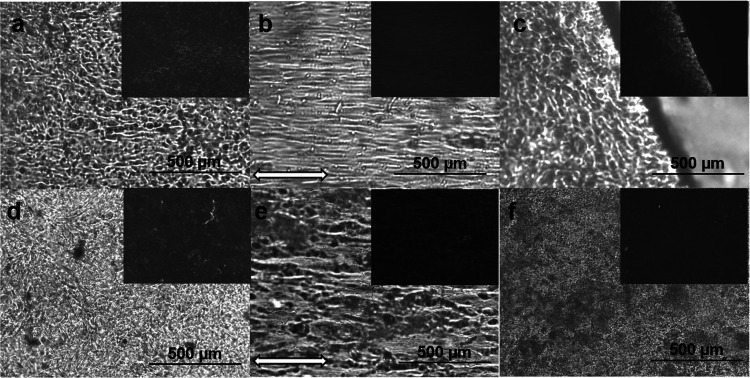
Optical microscopy images of PU/CNF films (0.4 mm thickness) with 0.5 wt% Br-CNF as a polyol: (a) as is, (b) under ca. 300 % strain (direction shown by the arrow), and (c) fractured edge; with 1.8 wt% Br-CNF as a chain extender: (d) as is, (e) under ca. 300 % strain (direction shown by the arrow); and with 5.4 wt% Br-CNF as a lone extender: (f) as is. Upper right insets were corresponding images under cross-polar mode.

Films with 0.5 wt% Br-CNF as a polyol and 1.8 wt% Br-CNF as an extender were uniaxially stretched at up to 300 % strain to observe their morphology by optical microscopy (Figure 7). The phase-separated hard (MDI-1,4-BD-MDI) and soft (PTMEG) microdomains appeared as granular black and white clusters in the PU control (Figure S5a), whereas PU containing either 0.5 wt% Br-CNF as a polyol or 1.8 wt% Br-CNF as a chain extender displayed isotropically arranged microfibers, under both transmission and cross-polar modes (Figure 7a,d). The same microfibers were also observed with 0.1 and 0.3 wt% Br-CNF as polyols (Figure S5b,c). The presence of microfibers illustrated inter-fibril Br-CNF association possibly by hydrogen bonding. Upon uniaxial stretching, the microfibers appeared to align along the loading direction from strain-induced stress stiffening above 200% (Figure S4). All microfibers were reoriented along the loading direction at 300 % strain (Figure 7b,e) and returned to the original isotropic arrangement (Figure 7a,d) upon unloading. The observed full reversibility for Br-CNF microfibers from isotropic (Figure 7a,d) to oriented alignment (Figure 7b,c) upon uniaxial stretching and then back to isotropic (Figure 7a,d) upon returning to zero strain demonstrated apparent elastic behavior at up to 300 % strain, unlike the inelastic stress relaxation observed at 400 % strain (Figure 6). The film fractured at 539 % strain also showed isotropic fibrils at the fracture edge (Figure 7c), indicative of the full reversibility of the PU/CNF film after releasing loading force even in the fracture region. The strain-induced fiber realignment in PU with Br-CNF as an extender (Figure 7f) was not as clear as that with Br-CNF as a polyol, supportive of the polyol role to be more effective in reinforcing PU.

## Conclusions

The stoichiometrically rationalized strategies demonstrated here show for the first time that 2-bromopropionyl bromide esterified cellulose nanofibrils (Br-CNFs) facilely synthesized from one-pot esterification of cellulose with 2-bromopropionyl bromide (BPB) and in situ ultrasonication can serve a dual role to partially replace either chain extenders or polyols in the syntheses of polyurethanes. The substituted surface 2-bromopropionyl ester (3.2 mmol/g) endows Br-CNF with excellent DMF dispersibility, while the unsubstituted surface OHs (3.5 mmol/g) are highly reactive to methylene diphenyl diisocyanate (MDI). Most importantly, the uniquely anisotropic (4.6 nm thick, 29.3 nm wide, ca. 1 μm long) and dual surface functional Br-CNF significantly reduced the MDI content to 15.7 % with the use of longer polytetramethylene ether glycol (PTMEG, *M*_n_: 2900 Da) as the soft segment. As a polyol, replacing merely 1.8 mol% PTMEG OHs with the surface OHs of Br-CNF (0.3 wt%) significantly improved the respective elastic modulus, tensile strength, and strain by 3.2, 3.9, and 1.5 times to 8.3 MPa, 21.1 MPa, and 755 %. As a chain extender, replacing 11 mol% 1,4-butanediol OHs with the surface OHs of Br-CNF (1.8 wt%) also improved the respective tensile properties to 8.3 MPa, 26.7 MPa, and 883 %, 27% higher in strength and 17 % higher in modulus. However, 6 times Br-CNF was required in the role of a chain extender compared to that required in the role of a polyol prepolymer. In the role of a polyol prepolymer, 0.3 wt% Br-CNF of the PU synthesized is the lowest among all reported to date while requiring only half MDI. The experimental modulus exceeding those predicted by the Halpin–Tsai model gave evidence of the synergistic effectiveness of optimal covalent bonding of Br-CNF with MDI and hydrogen bonding between Br-CNF and PTMEG. Intriguingly, the complete reversibility of isotropic Br-CNF under zero strain to oriented microfibril alignment at 300 % strain extends the elastic recovery of PU beyond the typical yield point. The efficiently synthesized Br-CNFs with unique organic compatibility and reactivity endowed by the respective surface 2-bromopropionyl ester and hydroxyls have enabled a rationally designed and stoichiometric synthetic strategy for the synthesis of significantly stronger polyurethanes with 50 % less diisocyanate. The newly synthesized 2-bromopropionyl esterified Br-CNFs offer novel synthetic strategies to not only maximize their reinforcing effect on polyurethane synthesized but also demonstrate potential dual-reactant and cross-linking roles of this functionalized nanocellulose in potential syntheses of other polymers.

## Supporting Information Available

Representative AFM and TEM images for 2-bromopropionyl bromide esterified cellulose nanofibrils; ^1^H NMR spectra for determining the level of substitution (ρ); XRD spectra for determining crystallinity; representative strain/stress curves for Br-CNF-reinforced PU with no excess MDI; and optical microscopy images for Br-CNF (0–0.3 wt %) reinforced PU films (PDF)
