# Supplementary material for: 2-Bromopropionyl Esterified Cellulose Nanofibrils as Chain Extenders or Polyols in Stoichiometrically Optimized Syntheses of High-Strength Polyurethanes
Source: Biomacromolecules. 2022 Oct 6;23(11):4574–85. doi: 10.1021/acs.biomac.2c00747 (PMC9667498; doi:10.1021/acs.biomac.2c00747)
Supplement: Supplementary file 1 — bm2c00747_si_001.pdf [file bm2c00747_si_001.pdf]

High Strength Thermoplastic Polyurethane Synthesized with 2-Bromopropionyl Esterified Cellulose Nanofibrils as Chain extender or Polyol

Mengzhe Guo and You-Lo Hsieh

Biological and Agricultural Engineering and Chemical Engineering, University of California at Davis, Davis, California 95616-8722,

United States

Email: ylhsieh@ucdavis.edu; Tel: +1 530 752 084

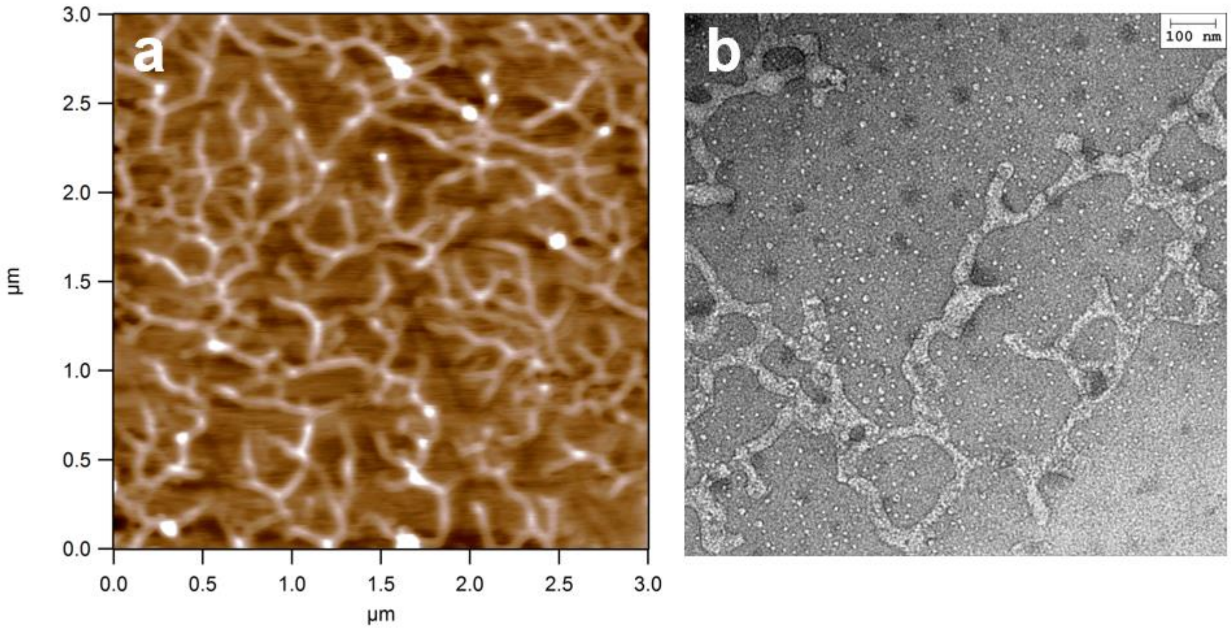

Figure S1. CNF Characteristics: (a) AFM height and (b) TEM images of CNFs.

The level of substitution (ρ) of Br-CNF by ^{1}H NMR. For each sample, 40 mL acetone was added to 10 mL Br-CNF dispersion in DMF (0.5 w/v%) and centrifuged (5k rpm, 10 min) to decant the supernatant, then repeated three times to prepare acetone gel. Br-CNF acetone gel (ca. 5 mg) was added into 1 mL DMSO-d_6, sonicated (10 min, Branson 2510) and vacuumed at 50°C for 1 h, then repeated three times to remove residual acetone. Br-CNF in DMSO-d_6 suspension was centrifuged (5k rpm, 10 min) and supernatant was collected for ^{1}H NMR (Bruker AVIII 600 MHz ^{1}H NMR spectrometer) characterization. Trifluoroacetic acid (50 µL) was added to DMSO-d_6 dispersion to shift all OHs peak downfield.

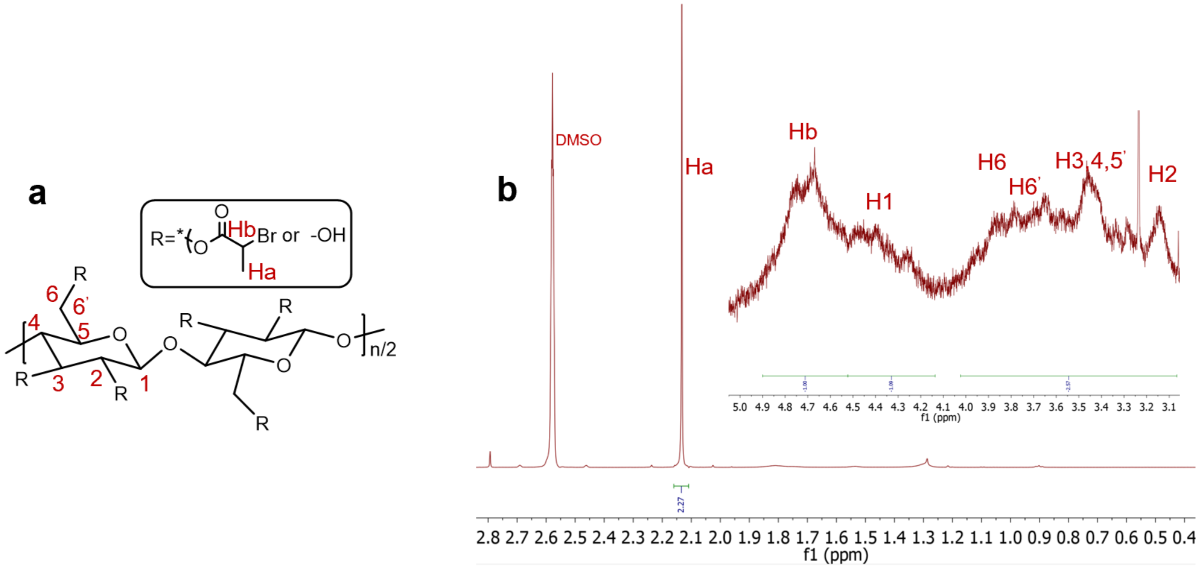

Figure S2. Br-CNF: (a) Structure and (b) ^{1}H NMR spectra.

The cellulose anomeric proton was the sum of the integrated areas for all anomeric H1 to H6' proton peaks averaged by 7. Br bearing esters were estimated by integration of the areas of methyl Ha divided by the respective 3 protons. The ratio of esterified C2, C3 and C6 OHs per surface AGU could be determined mathematically by the area ratio of Br ester calculated from Ha over the normalized anomic proton. The level of substitution (ρ), i.e., the fraction of OH substituted by

Br bearing ester as determined by proton Ha, was calculated by dividing ratio of esterified OHs per surface AGU by 3, i.e., 3 OH per AGU to be 48 %:

$$\rho = \frac{1}{3} \times \frac{\text{integral of methyl protons (Ha, doublet)}/3}{\sum_{1}^{6'} \text{integral of anomeric protons } (\text{H}^{\text{i}})\ /7} \tag{1}$$

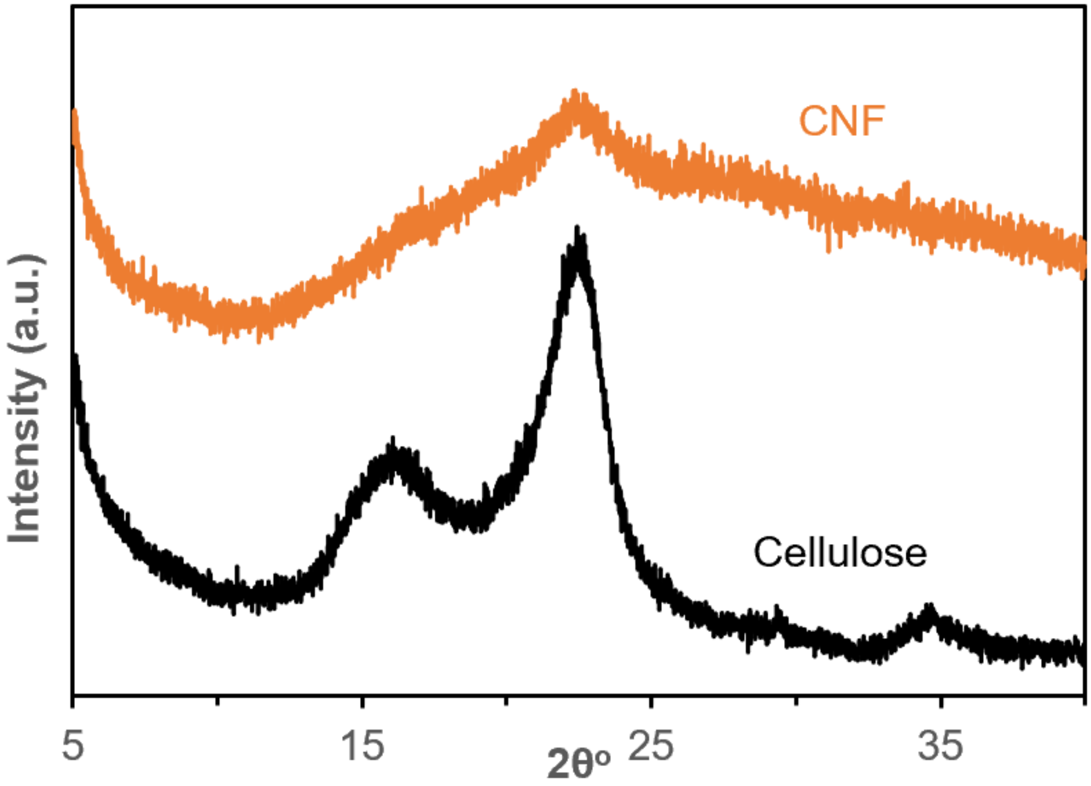

Figure S3. X-ray diffractograms of cellulose and Br-CNF.

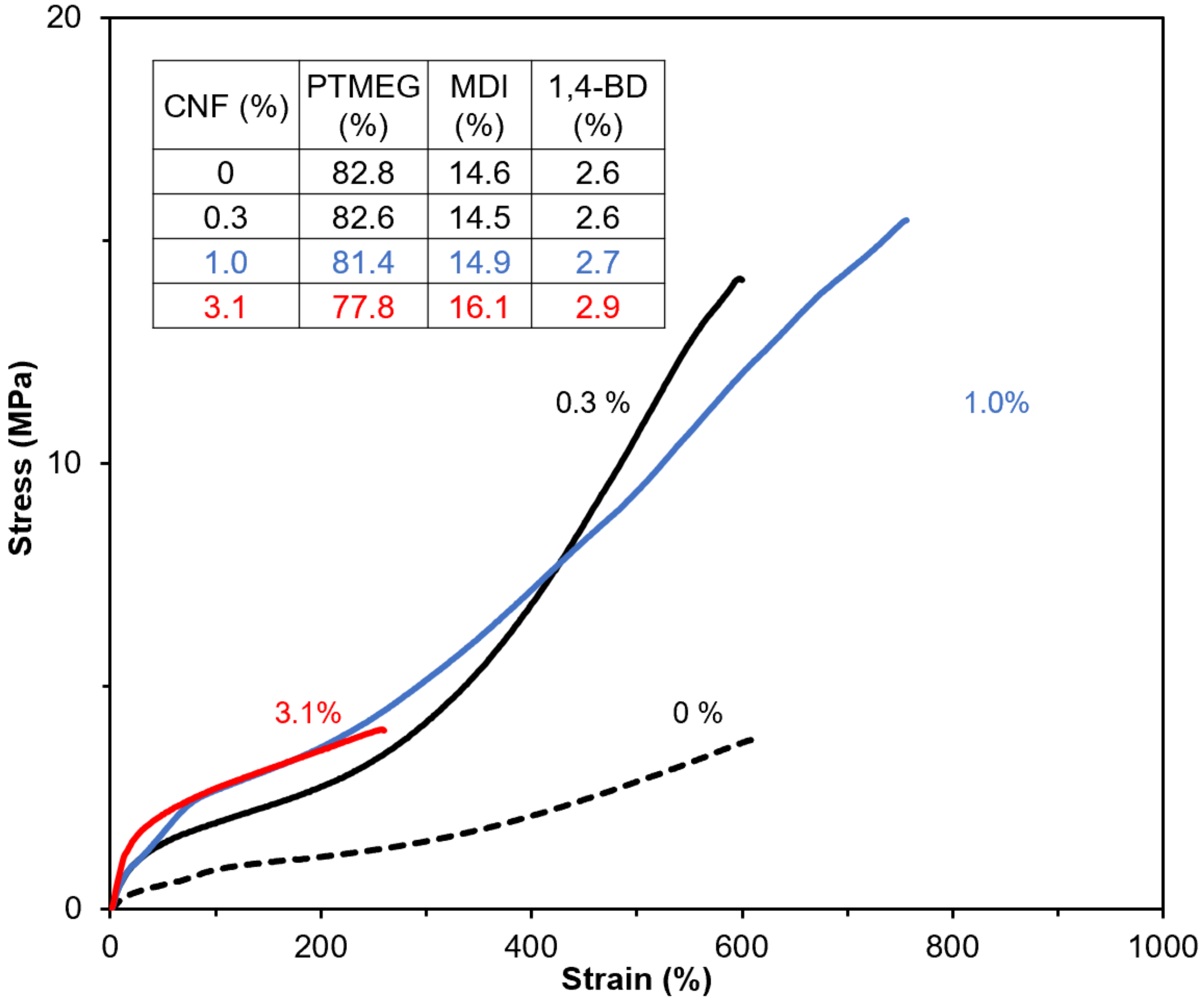

Figure S4. Stress-strain curves of PU synthesized with 0, 0.3, 1.0 or 3.1 % Br-CNF polyol with PTMEG (M_n=2,900*Da*) diol in 2:1:1 NCO_{MDI}: $OH_{PTMEG+CNF}$: $OH_{1,4\text{-}BD}$.

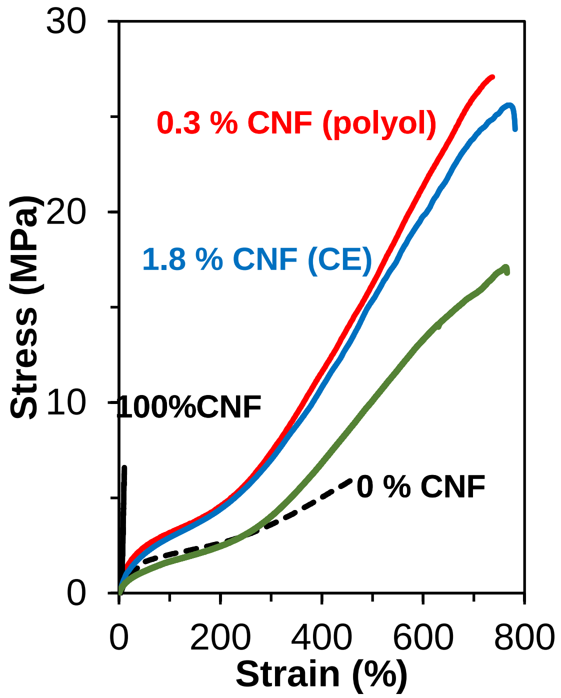

Figure S5. Stress-strain curves of PU synthesized with Br-CNF to partially replace both soft segment and chain extender, i.e., 1.8 mol% PTMEG (M_n=2,900*Da*) diol and 11 mol% EG chain extender, in 2:1:1 NCO_{MDI}: $OH_{PTMEG+Br\text{-}CNF}$: $OH_{EG\text{-}Br\text{-}CNF}$.

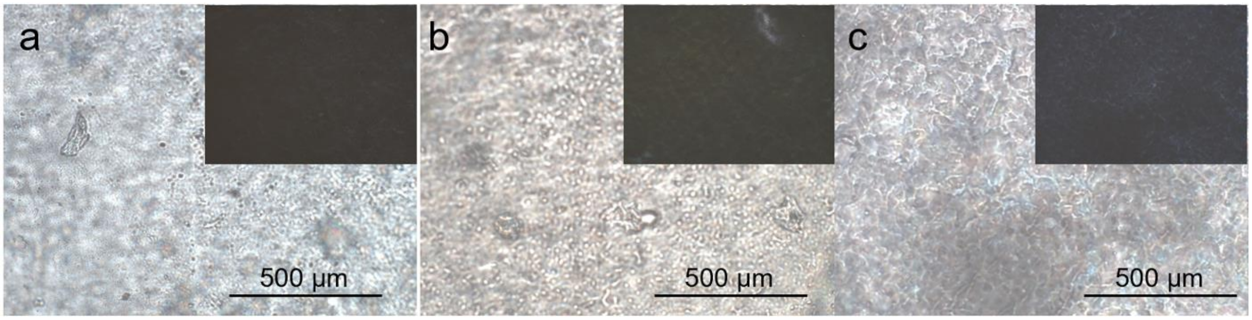

Figure S6. Optical microscopy images (10x) of upstretched PU films with (a) 0%, (b) 0.1%, (c) 0.3% Br-CNF as polyol. Insets on upper were corresponding images under cross-polar.
